# Dosimetric comparison of deformable image registration and synthetic CT generation based on CBCT images for organs at risk in cervical cancer radiotherapy

**DOI:** 10.1186/s13014-022-02191-3

**Published:** 2023-01-05

**Authors:** Yankui Chang, Yongguang Liang, Bo Yang, Jie Qiu, Xi Pei, Xie George Xu

**Affiliations:** 1 School of Nuclear Science and Technology, University of Science and Technology of China, Hefei, China; 2 Department of Radiation Oncology, Chinese Academy of Medical Sciences, Peking Union Medical College Hospital, Beijing, China; 3 Technology Development Department, Anhui Wisdom Technology Co., Ltd., Hefei, China; 4 Department of Radiation Oncology, First Affiliated Hospital of University of Science and Technology of China, Hefei, China

**Keywords:** CBCT, Deformable image registration, sCT generation, Dosimetric comparison, Cervical cancer

## Abstract

**Objective:**

Anatomical variations existing in cervical cancer radiotherapy treatment can be monitored by cone-beam computed tomography (CBCT) images. Deformable image registration (DIR) from planning CT (pCT) to CBCT images and synthetic CT (sCT) image generation based on CBCT are two methods for improving the quality of CBCT images. This study aims to compare the accuracy of these two approaches geometrically and dosimetrically in cervical cancer radiotherapy.

**Methods:**

In this study, 40 paired pCT-CBCT images were collected to evaluate the accuracy of DIR and sCT generation. The DIR method was based on a 3D multistage registration network that was trained with 150 paired pCT-CBCT images, and the sCT generation method was performed based on a 2D cycle-consistent adversarial network (CycleGAN) with 6000 paired pCT-CBCT slices for training. Then, the doses were recalculated with the CBCT, pCT, deformed pCT (dpCT) and sCT images by a GPU-based Monte Carlo dose code, ArcherQA, to obtain Dose_CBCT_, Dose_pCT_, Dose_dpCT_ and Dose_sCT_. Organs at risk (OARs) included small intestine, rectum, bladder, spinal cord, femoral heads and bone marrow, CBCT and pCT contours were delineated manually, dpCT contours were propagated through deformation vector fields, sCT contours were auto-segmented and corrected manually.

**Results:**

The global gamma pass rate of Dose_sCT_ and Dose_dpCT_ was 99.66% ± 0.34%, while that of Dose_CBCT_ and Dose_dpCT_ was 85.92% ± 7.56% at the 1%/1 mm criterion and a low-dose threshold of 10%. Based on Dose_dpCT_ as uniform dose distribution, there were comparable errors in femoral heads and bone marrow for the dpCT and sCT contours compared with CBCT contours, while sCT contours had lower errors in small intestine, rectum, bladder and spinal cord, especially for those with large volume difference of pCT and CBCT.

**Conclusions:**

For cervical cancer radiotherapy, the DIR method and sCT generation could produce similar precise dose distributions, but sCT contours had higher accuracy when the difference in planning CT and CBCT was large.

## Introduction

Cone-beam computed tomography (CBCT) images are widely used in image-guided radiation therapy (IGRT) systems, as they can monitor anatomical variations in clinical treatment. Since anatomical changes during radiotherapy treatment can alter the planned dose distribution [1, 2], dose recalculations based on CBCT images are necessary to obtain the fractional dose distribution for patients during radiotherapy. Unfortunately, radiotherapy dose calculations based on CBCT images can be inaccurate due to scattering and other artifacts [3–5], and so clinically, CBCT images are mostly utilized during pretreatment imaging for setup verification [6, 7]. Researchers are constantly striving to develop methods that can improve CBCT image quality, such as the iterative cone beam CT (iCBCT) of the Varian company, which combines statistical reconstruction and the Acuros CTS scattering correction algorithm [8, 9] to achieve uniform imaging with less noise and higher quality. Jarema et al. [10] validated the use of iCBCT for dose calculation in pelvis radiotherapy. Nevertheless, artifacts (cavity artifacts, etc.) still exist in iCBCT images, reducing the image structure recognition and dose calculation accuracy. Further CBCT applications in radiotherapy, such as adaptive radiotherapy, are similarly limited.

Various scholars have proposed methods to minimize dose calculation inaccuracies, which can be roughly classified into two types: projection domain methods and image domain methods. Projection domain methods suppress scatter during projection data acquisition, which can improve CBCT image quality by optimizing projection data [11, 12]. Image domain methods improve image quality with image processing algorithms [13–18], which can further be divided into 4 main categories: (1) calibration curve plotting between the HU and density (HU-D curve), the most commonly used in the clinic, which can be used to convert CBCT HUs to densities for dose calculation [19]; (2) the density assignment method (DAM), which segments an image into several tissue classes and assigns a suitable density to each class [16]; (3) deformable image registration (DIR) from CT to CBCT, in which the deformed CT images approximately represent the anatomical structures of the CBCT images, and the HU values are sufficiently accurate for dose calculation [20, 21]; and (4) synthetic CT (sCT) generation, which provides HU values similar to those of CT and anatomical structures similar to those of CBCT [22–25]. However, the HU-D curve can be sensitive to artifacts and scattering, and DAM cannot reflect subtle structures. DIR and sCT generation are superior to the other two methods. Barateau et al. [26] compared the 4 methods for H&N radiotherapy and demonstrated that the DIR method and sCT generation appeared to be the most appealing CBCT-based dose calculation methods.

In our previous study [27], the accuracy of the image quality and the structural consistency with CBCT images were compared between the DIR method and sCT generation method. The results demonstrated that both DIR and sCT generation could effectively improve the image quality of CBCT images. Due to the anatomic differences between the planning CT (pCT) and CBCT, especially in bladder volume, the accuracy of DIR appeared unsatisfactory, and the sCT generated by CycleGAN showed better structural consistency with CBCT. On the other hand, the sCT was obtained from trained model parameters, which might produce some artificial structures inconsistent with the CBCT images. The inconsistent anatomy of deformed pCT (dpCT) and artificial structures of sCT may introduce dose calculation errors, which require further validation. In this paper, we implemented dose recalculations on CBCT, pCT, dpCT and sCT images and compared the accuracy of the sCT dose and dpCT dose dosimetrically. Furthermore, the dosimetric accuracy of dpCT contours and sCT contours was analyzed compared with CBCT contours.

## Materials and methods

### Data acquisition

**Fig. 1:**
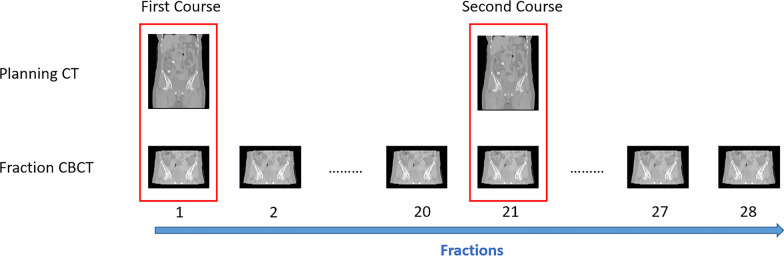
Data acquisition of paired CT and CBCT images. The data in the red box were collected in this study

A total of 190 paired CT and CBCT images from 115 cervical cancer patients were collected for retrospective analysis in this study, all of whom were treated with two-course intensity-modulated radiotherapy (IMRT): 20 fractions for the first course and 8 fractions for the second course. Of these 115 patients, 17 patients are in stage I, 43 patients in stage II, 52 patients in stage III and 3 patients in stage 4 (FIGO 2018). The total prescription dose was 50.4 Gy with 1.8 Gy/fraction, the patients who had finished the treatment had two planning CTs. Because there were still patients who had not finished the treatment when we collected the data, so such patients had one planning CT. Then, the paired CBCT was collected in the first fraction as shown in Fig. 1. A total of 150 paired CT-CBCT images were used for training the DIR network and sCT generation network, and the remaining 40 paired images were arranged for the dosimetric comparison.

The CT images were obtained on a PHILIPS BrillianceTM Bigbore CT, which has a bore with a diameter of 85 cm. The plane resolution of the CT ranged from 0.962 mm × 0.962 mm to 1.365 mm × 1.365 mm, and the slice thickness was 5 mm. The CBCT images were obtained from a Halcyon 2.0 system (Varian, USA), with plane resolution ranging from 0.908 mm × 0.908 mm to 1.035 mm × 1.035 mm and slice thickness of 2 mm. The range of the CBCT images was mainly concentrated near the clinical tumor target area, with a length of approximately 240 mm. The scanning range of the CT scan was longer than and completely overlapped that of the CBCT scan.

### Deformable image registration and sCT generation

The methods underlying the development of the DIR network and sCT generation network are described in detail in our previous work [27]. Briefly, the DIR network is based on a 3D multistage registration network (MSnet), which includes three stages of registration, each of which consists of two down-sampling layers, six ResNet blocks [28] and two upsampling layers. The MSnet model was trained and tested on Nvidia Geforce RTX 3090. The batch was set to 20 when the model in stage1, 4 in stage2, and 1 in stage3. The training required approximately 24 hours for 200 epochs. The sCT generation network is based on the 2D cycle-consistent generative adversarial network (CycleGAN), which mainly consists of two generators (G_CBCT-CT_, G_CT-CBCT_) and two discriminators (D_CT_, D_CBCT_): G_CBCT-CT_ generates the sCT images from the CBCT images, G_CT-CBCT_ generates the sCBCT images from the CT images, D_CT_ identifies the sCT images from the real CT images, and D_CBCT_ identifies the sCBCT images from the real CBCT images. ResNet with 15 ResNet blocks is used as the generator. sCT images are generated by G_CBCT-CT_ with CBCT images. The CycleGAN model was trained and tested on Nvidia Geforce RTX 3090. Adam was selected as the model optimizer. The batch was set to 6, the initial learning rate was set to 0.002 and the GAN discrimination rate was set to 0.02. The epoch number was set to 200 and the learning rate decreased linearly from 0.002 to 0 in last 100 epochs.

### Image processing

**Fig. 2:**
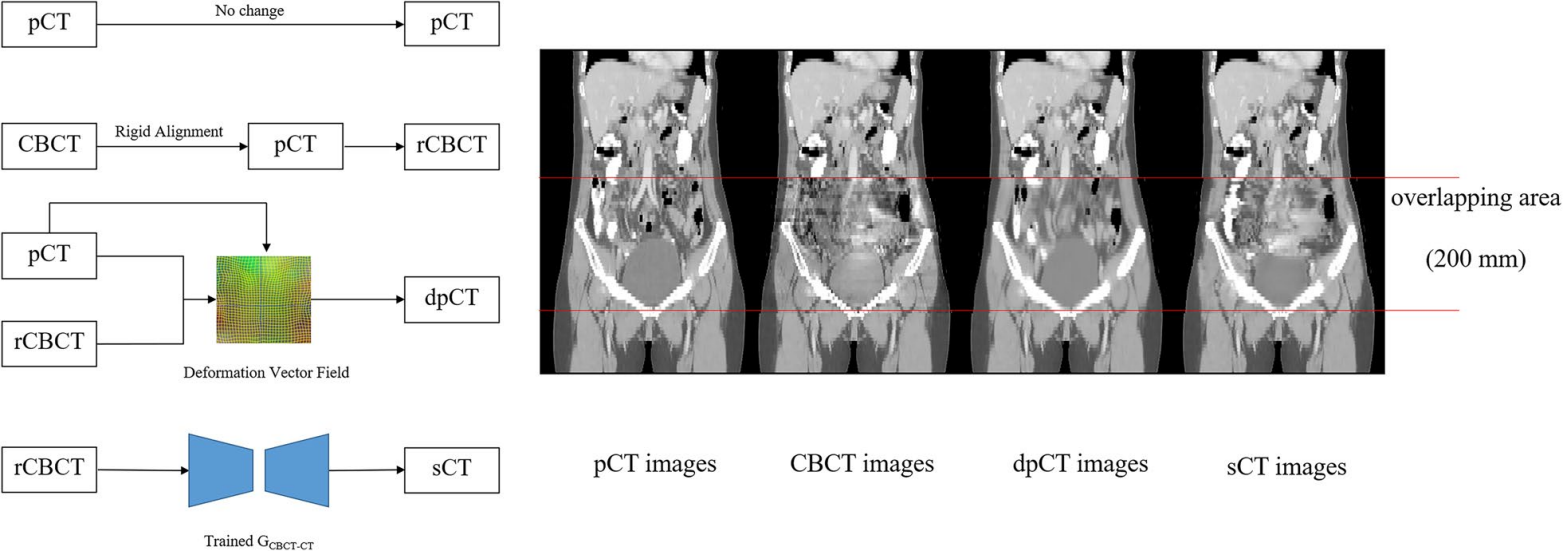
Pipeline and visualization of the synthesis of the pCT, CBCT, dpCT and sCT images. pCT: planning CT; dpCT: deformed planning CT; sCT: synthetic CT

As mentioned above, it was difficult to expand the scanning range of CBCT to cover the entire treatment area; the missing body data could affect the accuracy of dose calculation if it was directly used, and so the area not scanned by CBCT needed to be filled. As shown in Fig. 2, rigid alignment based on the Insight Toolkit (ITK) [29, 30] was first implemented from CBCT images to pCT images to obtain rigidly aligned CBCT images (rCBCT). In the process of rigid alignment, the coordinates and resolution of the CBCT images were made consistent with those of the pCT images. Because the information stored in the RP and RD files was based on the pCT images, the rigid alignment of CBCT images could better serve the dose calculation. Second, the dpCT images were obtained by the trained MSnet, and the sCT images were obtained by the trained generator G_CBCT-CT_. Finally, the area of overlap between the CBCT and pCT images was identified and marked. Outside the overlapping area, the pCT images were copied directly without any change. Within the overlapping area, the CBCT, dpCT or sCT images were used. It is worth mentioning that the field of vision (FOV) gradually decreased at both ends of the CBCT image, so the CBCT images at both ends could not contain complete images of the layer. In the actual operation, the middle 200 mm area was considered the overlapping area even though the full length of the CBCT images was 240 mm.

### Delineation and dose calculation

Structure delineation of the pCT images (pCT contours) was completed by experienced senior physicians and used for clinical radiotherapy. The dpCT contours were propagated from the pCT images to the corresponding CBCT images through deformation vector fields and sCT contours were auto-segmented and corrected manually. For the test cases, the physician redelineated the contours based on the CBCT images (CBCT contours). The contours for comparison included 7 organs at risk (small intestine, rectum, bladder, spinal cord, left femoral head, right femoral head and bone marrow).

The pCT, CBCT, dpCT and sCT images of 40 test cases were obtained through the above data processing in Fig. 2, whose image parameters (image position patient, image spacing, etc.) were consistent with those of the pCT images except for the HU values. Using the RP and RD files optimized based on the pCT images, the dose recalculations were implemented by a GPU-accelerated Monte Carlo code, ArcherQA, previously developed by our group [31, 32]. ArcherQA integrates the GPU acceleration function to quickly and accurately calculate the dose distribution based on CT images. This allowed the pCT, CBCT, dpCT and sCT doses to be obtained quickly by ArcherQA (Dose_pCT_, Dose_CBCT_, Dose_dpCT_, Dose_sCT_).

### Dose comparison and metrics

The global 3D gamma analysis (using an absolute dose comparison and 10% low-dose threshold) was completed with PTW Verisoft software, version 5.1 (PTW, Frieburg, Germany), using the following criteria: 1%/1 mm, 2%/2 mm, 2%/3 mm, 3%/2 mm, and 3%/3 mm.

Additionally, the dose discrepancies of organs at risk were analyzed. When analyzing the dose metrics, the dose distribution and contour information are essential. Each of the testing cases had 4 sets of dose distributions (pCT, CBCT, dpCT, sCT) and 4 sets of corresponding contours. If the respective dose distribution and contour information are used to analyze the dose metrics, the results would not be comparable. Therefore, we designed two groups of experiments:

1. Using the CBCT contours as the uniform contour information, the dose metric errors of Dose_pCT_, Dose_CBCT_, and Dose_sCT_ were calculated and evaluated compared with Dose_dpCT_.
2. Using Dose_dpCT_ as the uniform dose distribution, the dose metric errors of the pCT, dpCT and sCT contours were calculated and evaluated compared with CBCT contours.

In this study, the organs for comparison included the bladder, spinal cord, left femoral head, right femoral head and bone marrow, and the dose metrics included Dmean (the mean dose inside the organ) and D2 (the dose received by 2% of the volume). Dose metric errors were calculated using the following formula:

$${\text{Dose}}\;{\text{metric}}\;{\text{error}} = \frac{{\left| {M_{{{\text{comparison}}}} - M_{ground\;truth} } \right|}}{{M_{ground\;truth} }} \times 100\%$$

*M* represents the dose metric, including Dmean and D2. $M_{ground\;truth}$ represents the ground truth value of the dose metric, and $M_{{{\text{comparison}}}}$ represents the value of the dose metric to be compared. The value of the dose metric error represents the percentage; the smaller the value is, the closer it is to the ground truth. Paired sample t tests were used to evaluate the statistical significance of all the dose-volume parameters.

## Results

### CBCT image quality improvement

**Fig. 3:**
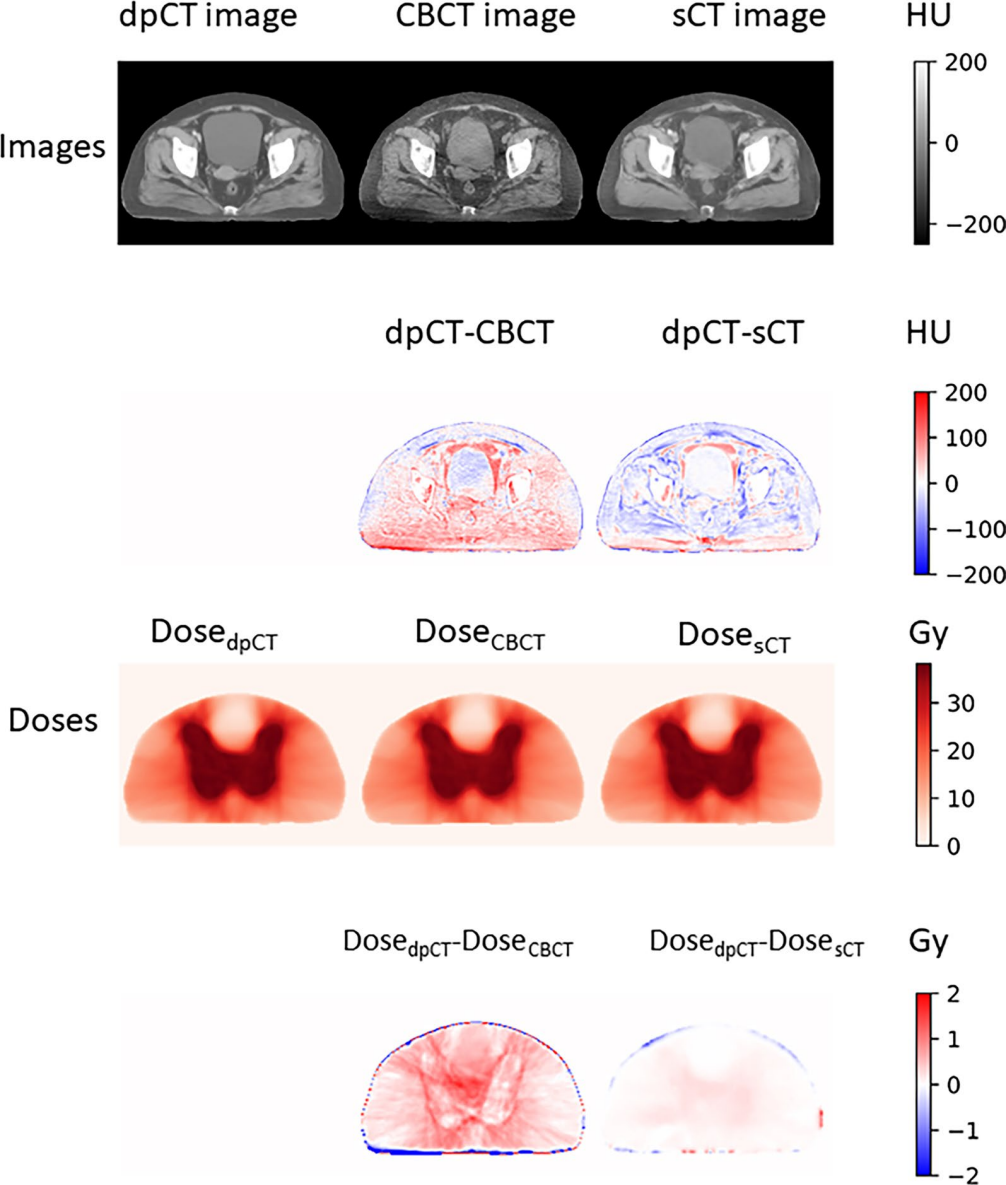
Visualized image differences and dose comparisons for dpCT, CBCT and sCT

Table 1 showed the gamma pass rates of Dose_CBCT_ and Dose_sCT_ using Dose_dpCT_ as the ground truth. The results in Table 1 indicated that Dose_sCT_ was highly consistent with Dose_dpCT_. Even at the criterion of 1%/1 mm, the average gamma pass rate of Dose_dpCT_ and Dose_sCT_ was 99.66%. Compared with those of Dose_CBCT_, the gamma pass rates of Dose_sCT_ were significantly improved at all criteria (1%/1 mm, 2%/2 mm, 2%/3 mm, 3%/2 mm, 3%/3 mm). Figure 3 showed the visualized image differences and dose comparisons for dpCT, CBCT and sCT. sCT had higher image quality and smaller dose calculation errors than CBCT.

**Table 1:** Comparison of gamma passing rates between Dose_CBCT_ and Dose_sCT_ (threshold = 10%, global 3D)

	1%/1 mm (%)	2%/2 mm (%)	2%/3 mm (%)	3%/2 mm (%)	3%/3 mm (%)
Dose_pCT_ vs. Dose_dpCT_	95.53 ± 3.53	99.12 ± 0.87	99.51 ± 0.61	99.52 ± 0.61	99.68 ± 0.47
Dose_CBCT_ vs. Dose_dpCT_	85.92 ± 7.56	97.92 ± 1.65	98.69 ± 1.24	99.31 ± 0.71	99.67 ± 0.52
Dose_sCT_ vs. Dose_dpCT_	99.66 ± 0.34	99.98 ± 0.05	99.99 ± 0.02	99.99 ± 0.02	100 ± 0
P^*^ value	*p* < 0.001	*p* < 0.001	*p* < 0.001	*p* < 0.001	*p* < 0.001

*P was calculated by comparing the gamma passing rates of Dose_CBCT_ vs. Dose_dpCT_ and Dose_sCT_ vs. Dose_dpCT_ according to paired sample t tests

### Accuracy of contours

The results in Table 2 showed the dice similarity coefficient (DSC) of pCT contours, dpCT contours and sCT contours using CBCT contours as ground truth. The accuracy of sCT contours was higher than the pCT and dpCT contours, especially in the deformable OARs (small intestine, rectum and bladder).

**Table 2:** DSC of pCT, dpCT and sCT contours

Contours	Small Intestine	Rectum	Bladder	Spinal Cord	Femoral Head L	Femoral Head R	Bone Marrow
pCT	0.842 ± 0.077	0.646 ± 0.119	0.733 ± 0.134	0.828 ± 0.117	0.870 ± 0.131	0.853 ± 0.162	0.804 ± 0.129
dpCT	0.844 ± 0.070	0.661 ± 0.119	0.756 ± 0.125	0.885 ± 0.065	0.917 ± 0.050	0.894 ± 0.115	0.883 ± 0.055
sCT	0.903 ± 0.054	0.789 ± 0.100	0.884 ± 0.072	0.936 ± 0.023	0.924 ± 0.012	0.911 ± 0.064	0.922 ± 0.011
P^*^ value	*p* < 0.001	*p* < 0.001	*p* < 0.001	*p* < 0.001	0.459	0.418	*p* < 0.001

*p-values were calculated by comparing DSC (sCT, CBCT) vs. DSC (dpCT, CBCT) according to paired sample t tests

### Dose metric analysis of organs at risk

**Fig. 4:**
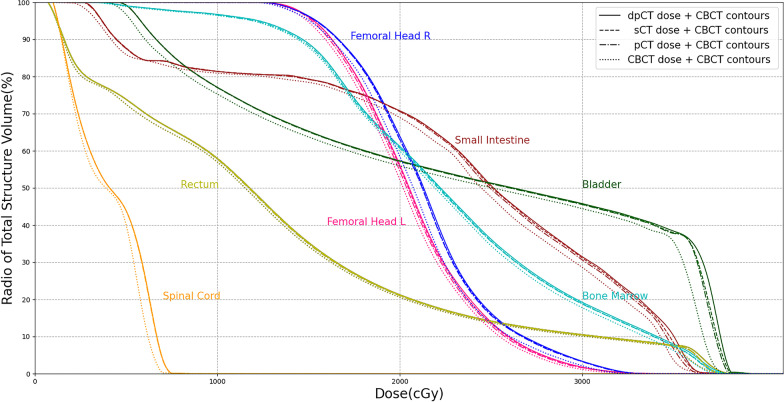
Dose-volume histograms with different dose distributions

Table 3 showed the dose metric errors for different dose distributions using the CBCT contours as the uniform contour information. The results showed that Dose_sCT_ had the smallest errors in Dmean and D2. The average dose metric errors of Dose_CBCT_ were in the range of 0.756–3.491%, the average dose metric errors of Dose_pCT_ were less than 0.8%, and the average dose metric errors of Dose_sCT_ were less than 0.3%. The results demonstrated the inaccuracy of CBCT-based dose calculation, which was greater than that caused by anatomical structures (Dose_pCT_ vs. Dose_dpCT_). Figure 4 showed the dose-volume histograms with different dose distributions for 9th paired data. The sCT-based curve and the dpCT-based curve almost completely overlapped, the pCT-based curve had small errors with the dpCT-based curve, and the CBCT-based curve had the largest deviation compared with the dpCT-based curve.

**Table 3:** Dose metric errors for different dose distributions using CBCT contours as the contour information (%)

Doses for comparison	Small intestine		Rectum		Bladder		Spinal Cord		Femoral Head L		Femoral Head R		Bone marrow	
	Dmean	D2	Dmean	D2	Dmean	D2	Dmean	D2	Dmean	D2	Dmean	D2	Dmean	D2
Dose_pCT_ vs. Dose_dpCT_	0.578	0.741	0.620	0.629	0.614	0.694	0.434	0.471	0.594	0.580	0.563	0.578	0.552	0.570
Dose_CBCT_ vs. Dose_dpCT_	1.229	0.756	1.998	1.384	2.488	1.765	3.491	2.767	1.673	1.537	1.830	1.736	1.395	1.320
Dose_sCT_ vs. Dose_dpCT_	0.186	0.257	0.145	0.222	0.243	0.271	0.074	0.122	0.173	0.221	0.161	0.187	0.162	0.208
P^*^ value	*p* < 0.001	*p* < 0.001	*p* < 0.001	*p* < 0.001	*p* < 0.001	*p* < 0.001	*p* < 0.001	*p* < 0.001	*p* < 0.001	*p* < 0.001	*p* < 0.001	*p* < 0.001	*p* < 0.001	*p* < 0.001

*P was calculated by comparing the dose metric errors of Dose_CBCT_ vs. Dose_dpCT_ and Dose_sCT_ vs. Dose_dpCT_ according to paired sample t tests

**Fig. 5:**
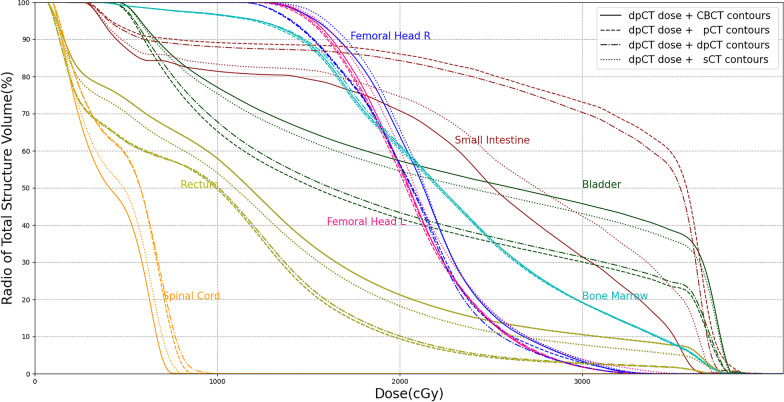
Dose-volume histograms with different contours

Table 4 showed the dose metric errors for different contours. Considering the anatomical structures and image quality, Dose_dpCT_ was used as the uniform dose distribution to analyze the dose errors caused by different contours. As shown in Table 4, the errors of both Dmean and D2 were smaller for rigid organs (femoral heads and bone marrow) and larger for deformable organs (bladder). We conducted further analysis for different volume changes using the difference in organ volume between pCT and CBCT as the analysis indicator. The 40 cases in the test set were divided into two groups, one with small Diff(V_CBCT_, V_pCT_) and the other with large Diff(V_CBCT_, V_pCT_). Table 4 shows that a larger Diff(V_CBCT_,V_pCT_) resulted in larger errors, especially for the pCT contours and dpCT contours. For the 20 cases with smaller Diff(V_CBCT_,V_pCT_), the sCT contours had lower errors, but none of the differences were significant (except for D2 of the spinal cord). For the 20 cases with larger Diff(V_CBCT_, V_pCT_), the sCT contours had comparable or lower errors, and significant differences were observed in the bladder. Figure 5 shows the dose-volume histograms with different contours for 9th paired data.

**Table 4:** Dose metric errors for different contours using dpCT-based dose distribution (%)

Category	Contours for comparison	Small Intestine		Rectum		Bladder		Spinal Cord		Femoral Head L		Femoral Head R		Bone Marrow	
		Dmean	D2	Dmean	D2	Dmean	D2	Dmean	D2	Dmean	D2	Dmean	D2	Dmean	D2
Smaller Diff(V_CBCT_,V_pCT_) (20 cases)	pCT contours vs. CBCT contours	1.443	8.166	0.687	3.907	0.314	3.400	4.972	2.659	1.957	3.446	2.064	3.559	0.744	1.540
	dpCT contours vs. CBCT contours	1.379	9.440	0.417	2.102	0.290	2.809	3.527	2.607	1.579	2.986	1.452	3.253	0.568	1.234
	sCT contours vs. CBCT contours	0.609	7.334	0.354	4.263	0.228	1.860	3.053	1.226	1.942	2.125	1.617	2.573	0.584	0.961
	P* value	0.018	0.455	0.754	0.116	0.638	0.123	0.647	0.013	0.296	0.191	0.694	0.430	0.908	0.419
Larger Diff(V_CBCT_,V_pCT_) (20 cases)	pCT contours vs. CBCT contours	2.991	13.634	1.004	9.730	0.389	6.517	6.834	2.891	2.122	3.548	1.983	4.110	0.985	2.426
	dpCT contours vs. CBCT contours	3.058	13.336	0.866	9.089	0.503	7.253	6.163	2.622	1.827	4.013	1.752	4.300	0.683	2.310
	sCT contours vs. CBCT contours	0.714	6.629	0.404	4.766	0.862	3.039	5.130	1.280	1.933	2.067	1.809	3.226	0.566	2.209
	P* value	0.001	< 0.001	0.166	0.051	0.536	0.011	0.242	0.014	0.640	0.128	0.902	0.381	0.510	0.714

*P was calculated by comparing the dose metric errors of the dpCT contours vs. CBCT contours and of the sCT contours vs. CBCT contours according to paired sample t tests

$$Diff(V_{CBCT} ,V_{pCT} ) = \frac{{|V_{CBCT} - V_{pCT} |}}{{MIN(V_{CBCT} ,V_{pCT} )}} \times 100\%$$

## Discussion

The results of this study suggested that both the DIR and sCT generation methods could yield improved calculation accuracies for doses based on CBCT images for cervical cancer patients. The iCBCT images in the Varian Halcyon 2.0 system are generated by combining statistical reconstruction and the Acuros CTS scattering correction algorithm, greatly improving the dose calculation accuracy. The average gamma pass rate at the 2%/2 mm criterion reached more than 97%. Figure 3 shows that errors persisted between the CBCT-based dose calculations and dpCT-based dose calculations, with many body voxel dose differences greater than 1 Gy. In contrast, the sCT-based dose calculations and dpCT-based dose calculations had higher consistency, and the gamma pass rate at the 1%/1 mm criterion was more than 99%. The consistency between Dose_sCT_ and Dose_dpCT_ illustrated not only the accuracy of sCT for dose calculation but also the accuracy of dose calculation based on dpCT, which was the major reason for using Dose_dpCT_ as the realistic dose distribution in fraction. Moazzezi et al. [33] also described that the scheduled dose was calculated on a simulated CT, which was produced by deformably registering the planning CT to the daily CBCT in Ethos. [34]

We considered a few reasons for the high consistency between the dpCT-based dose distribution and sCT-based dose distribution. The dpCT images were deformed from the pCT images to the CBCT images, and the sCT images were generated based on CBCT images, both of which had similar anatomical structures to the CBCT images, especially the high geometric similarity of the skin and bony structures. As we found in our previous work [27], the DIR method appeared to be ineffective for large deformable structures composed of soft tissue, and sCT might produce some artificial structures inconsistent with the CBCT images, which caused the slight difference in the anatomical structures of the dpCT images and sCT images. In Fig. 3, the sCT-based dose distribution was highly consistent with the dpCT-based dose distribution, which illustrated that the slight difference in the anatomical structures of the dpCT images and sCT images had little effect on the dose calculations. This result is in agreement with the conclusion of Liu et al. [35]. We believe that large deformable structures (bladder, etc.) were surrounded by other structures of similar HUs, even if the DIR method could not achieve high accuracy; when the HUs were converted into density according to the electron density curve, the small density difference resulted in a small dose difference. For sCT generation, most errors were in the vicinity of organs with similar HUs, which might result in more identification errors in anatomical structures but fewer errors in dose calculation.

Table 3 shows the dose metric errors for different dose distributions using uniform contours (CBCT contours). The results were roughly similar to those described in the above discussion: the sCT-based dose distribution had smaller errors, less than 0.3%, than the CBCT-based dose distribution. Table 4 shows that the sCT contours had smaller errors in the bladder and spinal cord, and there were comparable errors in the left femoral head, right femoral head and bone marrow for the pCT, dpCT and sCT contours. When the volume difference in pCT and CBCT images was large, sCT contours had higher accuracy in the deformable organs (small intestine, rectum and bladder). The combined analysis of Tables 3 and 4 showed that the errors caused by the different contours (Table 4) were larger than the errors caused by the different dose distributions (Table 3), which could be clearly seen from the comparison between Figs. 4 and 5. Our previous study [27] demonstrated that the DIR and sCT generation methods both improved the iCBCT image quality effectively, and sCT achieved higher accuracy when the difference between the planning CT and iCBCT was large. In this study, sCT produced a similarly precise dose distribution as dpCT, and the sCT contours had lower errors, demonstrating its clinical superiority over dpCT.

Some limitations should be noted in this study. First, the dosimetric analysis was carried out for 7 organs at risk because it was difficult to accurately identify the target on CBCT images. Second, the sCT contours could not be obtained with high accuracy automatically. Autosegmentation based on sCT images will be completed in our next work.

## Conclusions

We proposed two methods for improving CBCT image quality and compared the dose differences among planning CT, CBCT, deformed planning CT and synthetic CT images. The results showed that both the DIR method and the sCT generation method improved the dose calculation accuracy. The DIR method and sCT generation produced similarly precise dose distributions, with gamma pass rates of over 99% at the 1%/1 mm criterion and average dose metric errors less than 0.3%. In addition, the errors caused by the different contours were larger than the errors caused by the different dose distributions, and the sCT contours had lower errors and higher accuracy.

## Data Availability

The raw data supporting the conclusions of this article will be made available by the authors, without undue reservation.

## References

[1] Sonke JJ, Lebesque J, van Herk M (2008). Variability of four-dimensional computed tomography patient models. Int J Radiat Oncol Biol Phys.

[2] Kwint M (2014). Intra thoracic anatomical changes in lung cancer patients during the course of radiotherapy. Radiother Oncol.

[3] Marchant TE, Joshi KD, Moore CJ (2018). Accuracy of radiotherapy dose calculations based on cone-beam CT: comparison of deformable registration and image correction based methods. Phys Med Biol.

[4] Abe T (2017). Method for converting cone-beam CT values into Hounsfield units for radiation treatment planning. Int J Med Phys, Clin Eng Rad Oncol..

[5] Marchant TE, Joshi KD and Moore CJ. Shading correction for cone-beam CT in radiotherapy: validation of dose calculation accuracy using clinical images. In Proc. SPIE 10132, Medical Imaging 2017: Physics of Medical Imaging. 2017:10132;101320J1–101320J11.

[6] Pramanik S (2020). Analysis of setup uncertainties and determine the variation of the clinical target volume (CTV) to planning target volume (PTV) margin for various tumor sites treated with three-dimensional IGRT couch using KV-CBCT. J Radiat Oncol.

[7] Liu Z, Liu X, Zhang F (2018). How much margin do we need for pelvic lymph nodes irradiation in the era of IGRT?. Cancer.

[8] Maslowski A, Wang A, Sun M (2018). Acuros CTS: A fast, linear Boltzmann transport equation solver for computed tomography scatter - Part I: Core algorithms and validation. Med Phys.

[9] Wang A (2018). Acuros CTS: A fast, linear Boltzmann transport equation solver for computed tomography scatter: Part II: System modeling, scatter correction, and optimization. Med Phys.

[10] Jarema T, Aland T (2019). Using the iterative kV CBCT reconstruction on the Varian Halcyon linear accelerator for radiation therapy planning for pelvis patients. Phys Med.

[11] Lazos, D, Pokhrel, D, Zhong, S, et al. Experimental validation of a Monte Carlo-based kV x-ray projection model for the Varian linac-mounted Cone-Beam CT imaging system. In Medical Imaging 2008 - Physics of Medical Imaging. 2008. 6913

[12] Zhang Y (2020). Scatter correction based on adaptive photon path-based Monte Carlo simulation method in multi-GPU platform. Comput Methods Programs Biomed.

[13] Richter A (2008). Investigation of the usability of conebeam CT data sets for dose calculation. Radiat Oncol.

[14] Fotina I, Hopfgartner J, Stock M (2012). Feasibility of CBCT-based dose calculation: comparative analysis of HU adjustment techniques. Radiother Oncol.

[15] Yoo S, Yin FF (2006). Dosimetric feasibility of cone-beam CT-based treatment planning compared to CT-based treatment planning. Int J Radiat Oncol.

[16] Barateau A (2019). A density assignment method for dose monitoring in head-and-neck radiotherapy. Strahlenther Onkol.

[17] Giacometti V (2019). An evaluation of techniques for dose calculation on cone beam computed tomography. Br J Radiol.

[18] Li Y (2019). A preliminary study of using a deep convolution neural network to generate synthesized CT images based on CBCT for adaptive radiotherapy of nasopharyngeal carcinoma. Phys Med Biol.

[19] Schulze R (2011). Artefacts in CBCT: a review. Dentomaxillofac Radiol.

[20] Veiga C, McClelland J, Moinuddin S (2014). Toward adaptive radiotherapy for head and neck patients: feasibility study on using CT-to-CBCT deformable registration for ‘dose of the day’ calculations. Med Phys.

[21] Fu Y, Lei Y, Wang T (2020). Deep learning in medical image registration: a review. Phys Med Biol.

[22] Chen L, Liang X, Shen C (2021). Synthetic CT generation from CBCT images via unsupervised deep learning. Phys Med Biol.

[23] Deng L, Zhang M, Wang J, et al. Improving cone-beam CT quality using a cycle-residual connection with a dilated convolution-consistent generative adversarial network. Phys Med Biol. 2022. 10.1088/1361-6560/ac7b0a 35728794

[24] Thummerer A (2020). Comparison of CBCT based synthetic CT methods suitable for proton dose calculations in adaptive proton therapy. Phys Med Biol.

[25] Thummerer A (2020). Comparison of the suitability of CBCT- and MR-based synthetic CTs for daily adaptive proton therapy in head and neck patients. Phys Med Biol.

[26] Barateau A (2020). Comparison of CBCT-based dose calculation methods in head and neck cancer radiotherapy: from Hounsfield unit to density calibration curve to deep learning. Med Phys.

[27] Yang B (2022). A Comparison Study Between CNNBased Deformed Planning CT and CycleGAN-Based Synthetic CT Methods for Improving iCBCT Image Quality. Front Oncol.

[28] He K, Zhang X, Ren S (2016). Deep residual learning for image recognition. IEEE conference on computer vision and pattern recognition (CVPR)..

[29] McCormick M, Liu X, Jomier J (2014). ITK: enabling reproducible research and open science. Front Neuroinform.

[30] Yoo TS at al. Engineering and Algorithm Design for an Image Processing API: A Technical Report on ITK – The Insight Toolkit. In Proc. of Medicine Meets Virtual Reality, J. Westwood, ed., IOS Press Amsterdam. 2002. 586–592 15458157

[31] Su L (2014). ARCHERRT—a photon-electron coupled Monte Carlo dose computing engine for GPU: software development and application to helical tomotherapy. Med Phys.

[32] Peng Z (2022). Development of a GPU-accelerated Monte Carlo dose calculation module for nuclear medicine, ARCHER-NM: demonstration for a PET/CT imaging procedure. Phys Med Biol.

[33] Moazzezi M, Rose B, Kisling K (2011). Prospects for daily online adaptive radiotherapy via ethos for prostate cancer patients without nodal involvement using unedited CBCT auto-segmentation. J Appl Clin Med Phys.

[34] Archambault Y (2020). Making on-line adaptive radiotherapy possible using artificial intelligence and machine learning for efficient daily re-planning. Med Phys Intl J.

[35] Liu Y (2020). CBCT-based synthetic CT generation using deep-attention cycleGAN for pancreatic adaptive radiotherapy. Med Phys.

